# Vogt–Koyanagi–Harada disease with a unilateral presentation in a patient with marijuana overuse: Role of multimodal imaging in suspected patients

**DOI:** 10.1002/ccr3.7879

**Published:** 2023-09-04

**Authors:** Seyedeh Maryam Hosseini, Ahmad Gharouni, Mehrdad Motamed Shariati

**Affiliations:** ^1^ Eye Research Center, Mashhad University of Medical Sciences Mashhad Iran

**Keywords:** inflammation, multimodal imaging, tetrahydrocannabinol, Vogt–Koyanagi–Harada disease

## Abstract

**Key Clinical Message:**

Harada disease could uniquely present with only unilateral symptoms, as was seen in our patient. However, multimodal imaging including ICG angiography could show bilateral involvement. Considering the immunomodulatory effects of Cannabis, the absence of inflammatory findings and the unusual presentation of the disease, in our case, may have been caused by the use of marijuana.

**Abstract:**

To report a patient addicted to marijuana with the diagnosis of Vogt–Koyanagi–Harada (VKH) disease with a unilateral presentation. A 24‐year‐old man presented to us with painless decreased vision in his right eye (RE) and photophobia 3 days ago. No history of significant family or past medical history was documented. Spectral‐domain optical coherence tomography (SD‐OCT) of the RE showed multiple areas of subretinal fluid in the macula. Indocyanine green angiography (ICGA) revealed round hypocyanescent dark dots (HDD) of similar size, evenly distributed in both eyes. With the diagnosis of VKH disease, anti‐inflammatory treatment was started. To our knowledge, this is the first reported case of suspected VKH in a patient with marijuana overuse. Regarding the complex effects of tetrahydrocannabinol (THC), the active ingredient of marijuana, on the vascular and immune systems, reaching a definite conclusion is not possible. This report shows the value of multimodal imaging in patients with unusual presentations.

## Graphical abstract

Multimodal imaging of the patient's right eye, which is compatible with Vogt‐Koyanagi‐Harada disease.

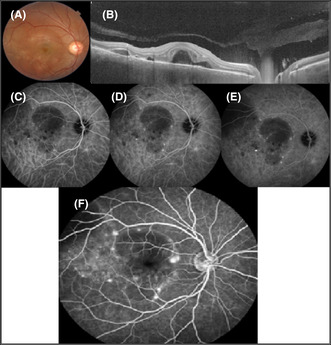

## 1 INTRODUCTION

Vogt–Koyanagi–Harada (VKH) disease is a chronic systemic granulomatous inflammation with neurologic, integumentary, auditory, and visual systems involved in different stages of the disease. Based on the extent of systemic involvement, this disease is classified into complete, incomplete, and probable VKH.^1^ Isolated ocular involvement in a patient with no history of penetrating ocular trauma or surgery preceding the initial onset of uveitis is defined as the probable VKH. Eye involvement typically occurs bilaterally, but evidence of the disease may occur in the second eye with a delay of 2–3 weeks.^2^

Early manifestations of ocular involvement included diffuse choroiditis, with or without anterior uveitis, vitreous inflammation, or optic nerve head hyperemia. In the late stages, fundus depigmentation, recurrent or chronic anterior uveitis, and pigment clumping may occur.^3^

Tetrahydrocannabinol (THC), the active ingredient of marijuana, relaxes arterial walls, increasing blood flow to tissues.^4^ In the brain, THC binds to ubiquitous cannabinoid receptors (CB1) in arterial tissue and regulates the microvascular environment via dose‐dependent dilation of cerebral arterioles.^5^ However, the effects of this psychoactive agent on choroidal blood flow are unknown. The immune modulatory effects of cannabis and its derivatives have been reported previously.^6^

In this report, we aimed to introduce a patient addicted to marijuana with the diagnosis of probable VKH with a unilateral presentation.

## 2 CASE REPORT

A 24‐year‐old man presented to us with painless decreased vision in his right eye (RE) and photophobia for the past 3 days. No history of significant family or past medical history was documented. The patient stated that he overused marijuana for 2 years.

On ocular examination, his best‐corrected visual acuity (BCVA) was 10/20 for the RE and 20/20 for the left eye (LE). The pupil examination revealed round and reactive pupils and a negative relative afferent pupillary defect.

Extraocular movements were normal. Intraocular pressure measured 13 mmHg for the RE and 14 mmHg for the LE. Anterior segment examination was unremarkable. We found multiple foci of subretinal fluid (SRF) at the macula of the RE. The optic nerve head (ONH) examination was normal. The examination of the LE was unremarkable.

Multimodal imaging including spectral‐domain optical coherence tomography (SD‐OCT) (Heidelberg Eye Explorer version 1.9.13.0, Spectralis Viewing Module 6.5.2.0; Heidelberg Engineering), fluorescein angiography (FAG) (Heidelberg Eye Explorer version 1.9.13.0, Spectralis Viewing Module 6.5.2.0; Heidelberg Engineering), and indocyanine green angiography (ICGA) (Heidelberg Eye Explorer version 1.9.13.0, Spectralis Viewing Module 6.5.2.0; Heidelberg Engineering) had been performed to make a more accurate diagnosis.

**FIGURE 1:**
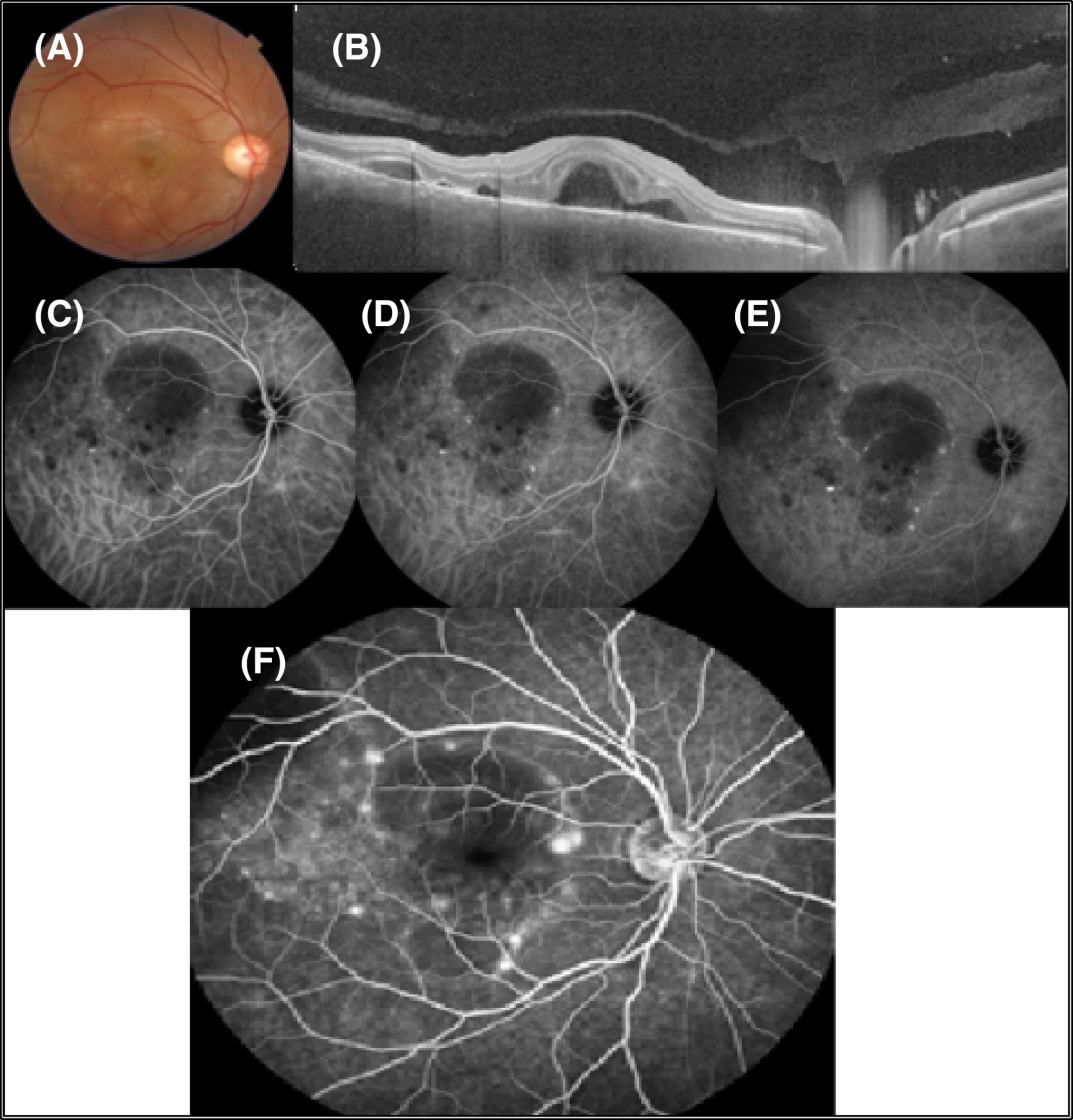
Multimodal imaging of the RE. Multiple foci of subretinal fluids (SRF) in macula with multiple areas of SRF and increased thickness of choroid are apparent in B‐scan OCT (A, B). Multiple hypocyanescence dark dots, a circular area of blocked fluorescence because of SRF, and enlarged and fuzzy choroidal blood vessels are visible in ICGA (C–E). Fluorescein angiography shows multiple pinpoint hyperfluorescence compatible with leakage (F).

**FIGURE 2:**
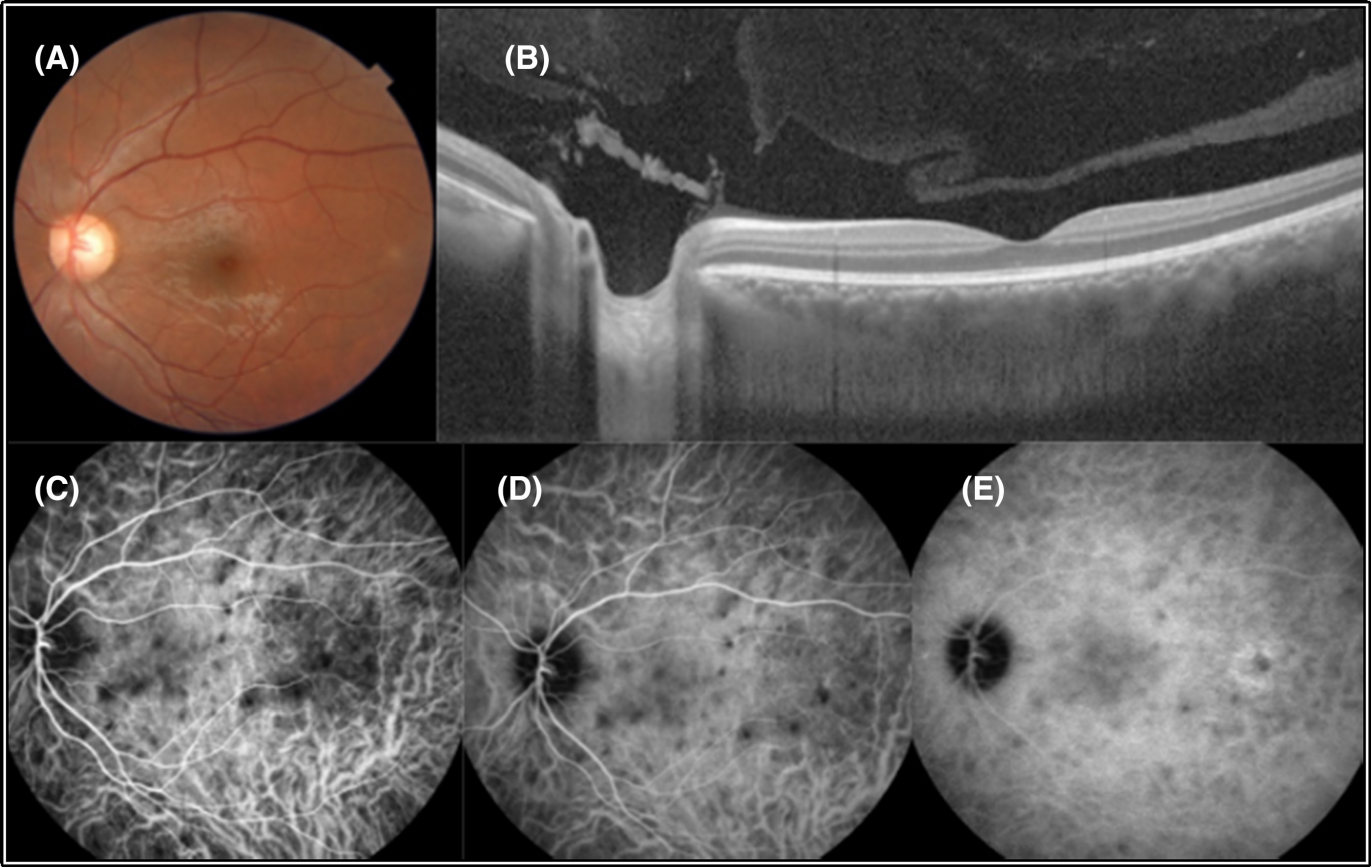
Multimodal imaging of the LE. No SRF is apparent in fundus photograph (A) and B‐scan OCT (B) of the patient. Multiple hypocyanescence dark dots, and enlarged and fuzzy choroidal blood vessels are visible in ICGA (C–E).

Spectral‐domain optical coherence tomography of the RE showed multiple areas of SRF in the macula. Furthermore, choroidal thickening was obvious in both eyes. Fluorescein angiography showed multiple foci of leakage compatible with the areas of SRF with the pooling of dye into the subretinal spaces in the RE (Figure 1). Indocyanine green angiography revealed round hypocyanescent dark dots (HDD) of similar size, evenly distributed in both eyes (Figures 1 and 2).

Regarding the patient's signs and imaging findings and no history of ocular trauma or surgery, the patient was hospitalized with the diagnosis of probable VKH. Systemic assessments including the PPD test, serum level of angiotensin‐converting enzyme (ACE) and chest X‐ray (CXR) had no remarkable results. Intravenous methylprednisolone (1 g/day) was started and continued for 3 days. The patient was discharged with partial recovery of his visual symptoms with oral prednisolone (50 mg/day). Immune modulatory therapy (IMT) was started (Mycophenolate mofetil 500 mg every 12 h).

**FIGURE 3:**
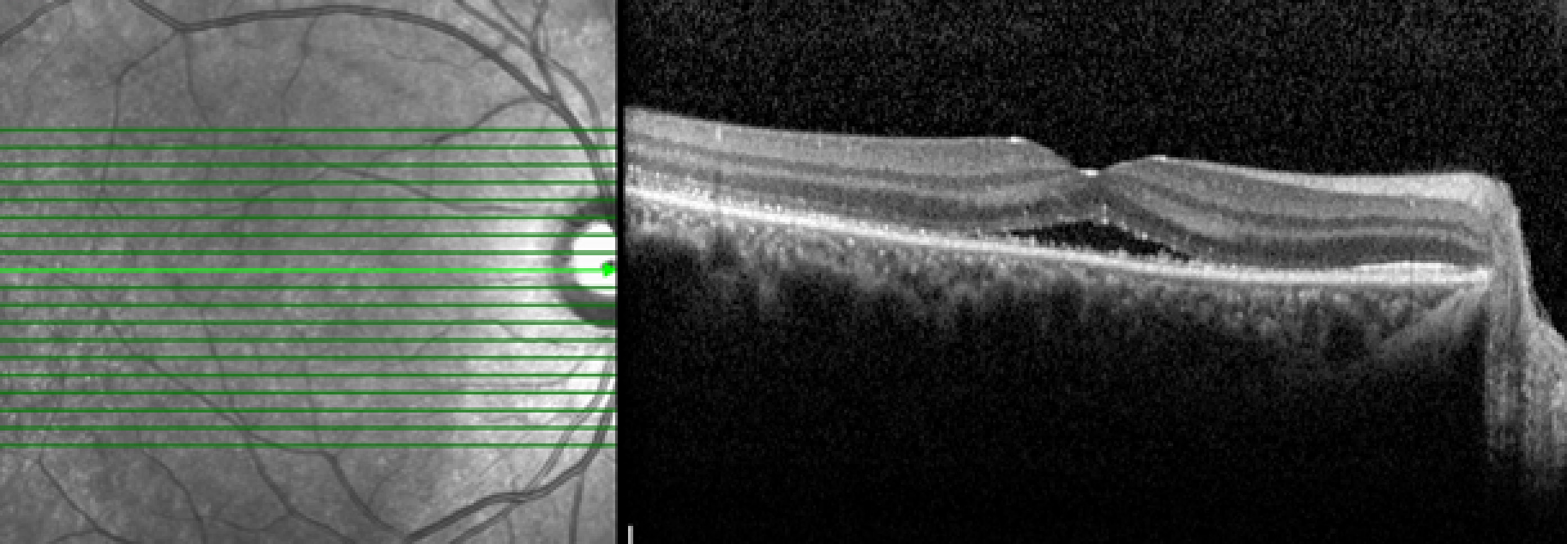
Infrared reflectance and B‐scan OCT images showed decreased SRF 1 month after the treatment initiated.

At the 1‐month follow‐up visit, the macular SRF was absorbed and the patient's visual acuity fully recovered to 20/20 (Figure 3). The patient is under medical treatment with mycophenolate to taper off the oral prednisolone very slowly for 6 months.

## 3 DISCUSSION

A wide range of disorders involving the choroid can lead to fluid accumulation in the subretinal space. Central serous chorioretinopathy (CSC), hypertensive choroidopathy, choroidal tumors, and choroidal inflammations are some examples of the differential diagnosis of SRF accumulation.^7^

In this article, we introduced a case of suspected VKH (Harada disease) with a unilateral presentation. Typically, VKH is a systemic granulomatous disease with bilateral ocular involvement. However, previous studies reported some cases of VKH with a unilateral presentation.^8^, ^9^ The main challenge in this patient is the lack of evidence of inflammation such as papillitis, vitreous haziness, and anterior segment inflammation. Besides, he overused marijuana. Tetrahydrocannabinol is a psychoactive agent that has some vasomotor effects including an increase in tissue blood flow.^6^ Although the effect of THC on the choroidal blood flow is unknown, choroidal hyperemia could potentially be a risk factor for SRF accumulation.

Considering the presence of photophobia and OCT findings and the absence of any underlying systemic disease, we decided to further evaluate the patient with FAG and ICGA. The multimodal imaging revealed stromal choroidopathy. Tuberculosis choroiditis, syphilis, sarcoidosis, uveal lymphoid proliferation, and posterior scleritis are the major differential diagnoses in our patient. Regarding the absence of a history of trauma, eye surgery, no remarkable finding in systemic evaluations, and the patient's clinical course, suspected VKH was raised as the final diagnosis.

**FIGURE 4:**
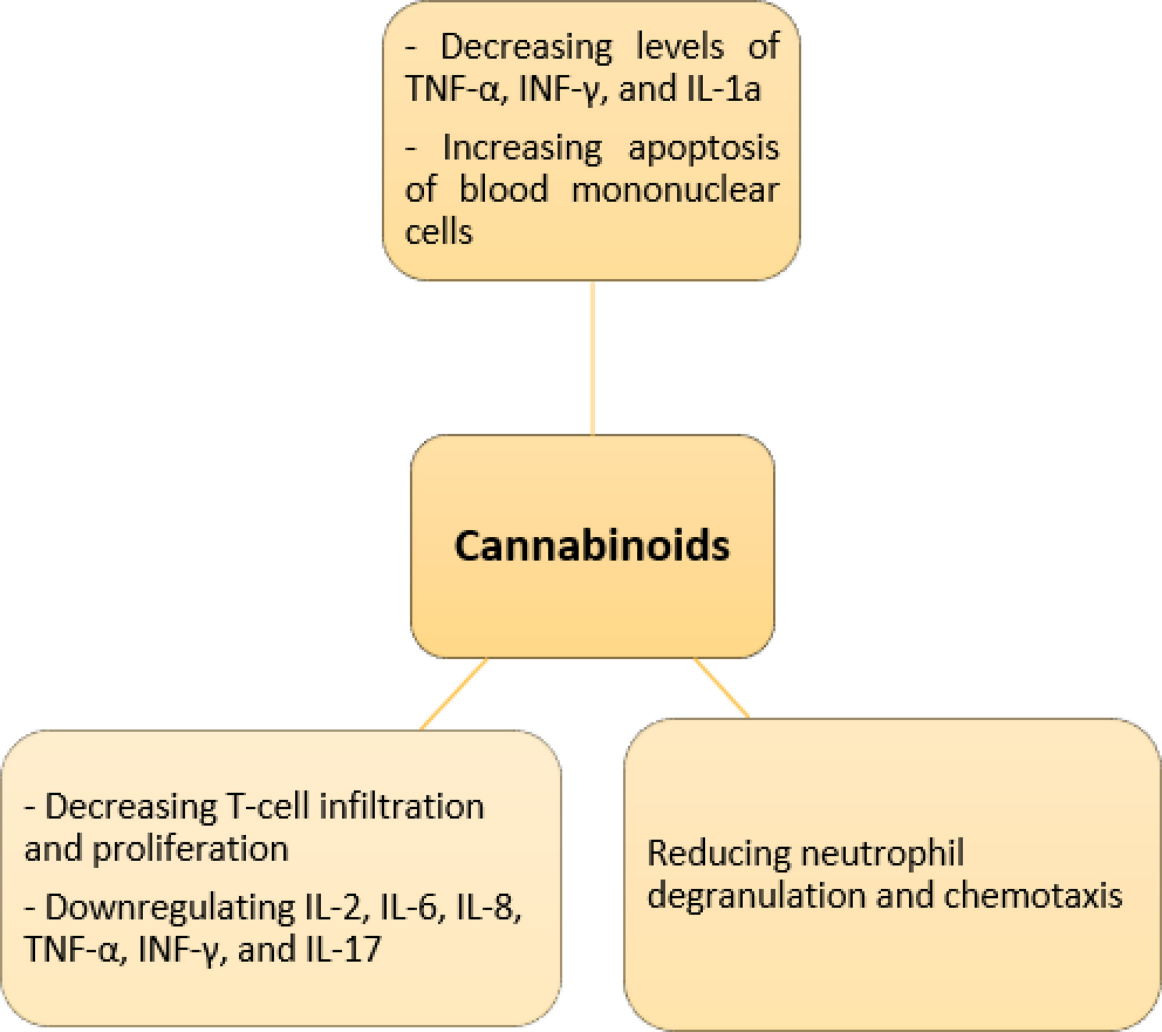
Anti‐inflammatory effects of cannabinoids.

Previous studies investigated the effects of cannabis and its derivatives on the immune system.^10^, ^11^ We summarized the anti‐inflammatory effects of cannabinoids in Figure 4. Stimulation of CB receptors can lead to the weakening of the immune system by reducing the immune cell migration, decreasing the production of inflammatory cytokines, and apoptosis induction.^6^ Previous studies suggest the role of the endocannabinoid system in autoimmune and inflammatory diseases such as rheumatoid arthritis (RA), multiple sclerosis (MS), and inflammatory bowel disease (IBD).^6^ The immunomodulating properties of cannabis have been shown in animal and in‐vitro studies,^12^, ^13^ although there is a lack of evidence and clinical studies on the therapeutic and side effects of this compound. We hypothesize that the absence of inflammatory findings and the unusual presentation of the disease, in this case, may have been caused by the use of marijuana.

## 4 CONCLUSION

To our knowledge, this is the first reported case of suspected VKH in a patient with marijuana use disorder. Regarding the complex effects of THC on the vascular and immune systems, reaching a definite conclusion is not possible. This report shows the value of multimodal imaging in patients with unusual presentations.

## AUTHOR CONTRIBUTIONS

**Seyedeh Maryam Hosseini:** Data curation; supervision; writing – review and editing. **Ahmad Gharouni:** Data curation; investigation. **Mehrdad Motamed Shariati:** Conceptualization; data curation; investigation; supervision; writing – original draft; writing – review and editing.

## FUNDING INFORMATION

The authors received no funding.

## CONFLICT OF INTEREST STATEMENT

The authors declare that they have no competing interests.

## CONSENT

Written informed consent was obtained from the patient to publish this report in accordance with the journal's patient consent policy.

## Data Availability

The datasets used during the current study are available from the corresponding author upon reasonable request.
